# Error-based and reinforcement learning in basketball free throw shooting

**DOI:** 10.1038/s41598-022-26568-2

**Published:** 2023-01-10

**Authors:** Charlène Truong, Célia Ruffino, Alexandre Crognier, Christos Paizis, Lionel Crognier, Charalambos Papaxanthis

**Affiliations:** 1 INSERM UMR1093-CAPS, Université Bourgogne Franche-Comté, UFR des Sciences du Sport, UFR STAPS, Campus Universitaire, BP 27877, 21000 Dijon, France; 2 Pôle Recherche et Santé Publique, CHU Dijon Bourgogne, 21000 Dijon, France; 3 INSERM, CIC 1432, CHU Dijon-Bourgogne, Centre d’Investigation Clinique, Module Plurithématique, Plateforme d’Investigation Technologique, 21000 Dijon, France; 4 Centre for Performance Expertise, Université Bourgogne Franche-Comté, UFR des Sciences du Sport, 21000 Dijon, France

**Keywords:** Learning and memory, Consolidation

## Abstract

This study investigates the effects of error-based and reinforcement training on the acquisition and long-term retention of free throw accuracy in basketball. Sixty participants were divided into four groups (n = 15 per group): (i) the error-based group (sensory feedback), (ii) the reinforcement group (binary feedback including success or failure), (iii) the mixed group (sensory feedback followed by binary feedback), and (iv) the control group (without training). Free throws success was recorded before training (PreT), immediately after (Postd0), one day later (Postd1), and seven days later (Postd7). The error-based group, but not the reinforcement group, showed a significant immediate improvement in free throw accuracy (PreT vs Postd0). Interestingly, over time (Postd0 vs Postd1 vs Postd7), the reinforcement group significantly improved its accuracy, while the error-based group decreased it, returning to the PreT level (PreT vs Post7). The mixed group showed the advantage of both training methods, i.e., fast acquisition and retention on a long-term scale. Error-based learning leads to better acquisition, while reinforcement learning leads to better retention. Therefore, the combination of both types of learning is more efficient for both acquisition and retention processes. These findings provide new insight into the acquisition and retention of a fundamental basketball skill in free throw shooting.

## Introduction

Accuracy is one of the most important components of numerous motor skills^1^. In several sports, such as basketball or archery, and professional activities, such as typing or driving, accuracy is essential to performance^1,2^. To improve movement accuracy, the most basic training method is e*rror-based* learning^3,4^. Simply saying, the error in a movement guides the next movement enhancing its accuracy; such as the trial-by-trial improvement during a free kicking practice in football^5^, a serving practice in volleyball^6^, and a free throw practice in basketball^7^.

The error-based learning process is currently supported by the theory of internal forward models^8,9^. Accordingly, in learning situations, the sensorimotor system predicts the sensory consequences (e.g., position and velocity) of the planned-intended movement and compares this prediction to the actual sensory feedback from its execution^10,11^. If there is no discrepancy, then the planned and executed movement is similar. If a difference is detected, it constitutes an error signal that informs how the movement failed (i.e., the knowledge of the error vector; for example, the basketball fell to the left or the right of the hoop), leading to the correction of motor command for the future attempt^12^. When the movement execution improves, the error signal decreases; thus, the error vector guides the learning process. Error-based learning is a powerful method leading to the rapid acquisition of motor skills^13^. Fundamental and clinical studies have shown that the cerebellum plays a critical role in sensorimotor prediction and thus in error-based learning^14–16^. Plastic mechanisms, changing the neural pathways between the cerebellum and the primary motor cortex, are engaged in error-based learning^17^.

Reinforcement is another method of motor learning^18^. It is based on simple feedback regarding movement success or failure, depriving thus the learner of sensory information about the appropriate correction to achieve the desired movement^17–19^. For example, a basketball learner is simply informed whether his/her free throw was successful or not. In this context, the learner has visual feedback neither of his/her movement nor the ball’s trajectory. Therefore, to attain a successful free throw, the solution lies in a vast motor exploration, namely a trial-and-error process searching for the best motor outcome. During this exploration, the desired reward prediction is compared to the actual reward and a success value is assigned to each movement^18^. A motor skill is acquired by selecting and reinforcing the appropriate action that maximizes success and avoids failure. While acquisition (i.e., immediate improvement performance) is slower in reinforcement learning than in error-based learning, long-term retention (i.e., consolidation) is much better^19–21^. Thus, in a basketball game context, the learner will be able to select the appropriate movement previously reinforced during reinforcement learning. It is well established that reinforcement learning involves neural pathways between the basal ganglia and the primary motor cortex^22,23^, which are facilitated by dopaminergic neurons^24,25^. In addition, it appears that reinforcement learning occurs naturally, especially when there is no more error in movement execution, leading to better retention^26^.

Feedback is important for motor skill learning, allowing identifying the correct performance and making the necessary adjustments^27–30^. The two above-described learning methods, requiring different types of feedback, have been mainly debated in experimentally constrained tasks in the laboratory (e.g., pointing and reaching tasks). As the human motor repertoire offers a wide range of possibilities, especially in the sports domain requiring whole body and complex movements^31^, the challenge of this study was to transfer the principles of motor learning from the laboratory to the sports field. For this purpose, the basketball free throw shooting movement was selected. This skill requires great accuracy and involves movement redundancy, due to the utilization of the entire body and multiple ball trajectories to score. Precisely, the motor memory formation of basketball free throw shooting was investigated immediately, one day, and seven days after error-based (training with sensory feedback) and reinforcement (training with only success or failure feedback) learning, as well as after the combination of these methods (mixed: error-based followed by reinforcement learning). To exclude a potential effect of simple movement repetition during the test sessions (i.e., before training, immediately, one day, and seven days after training) on memory formation, which could be confounded with a true training effect, we introduced a control condition (control group), without any training.

In light of the literature suggesting that error-based training leads to faster acquisition and reinforcement training leads to better retention^13,16,19–21,32^, we hypothesized that this pattern would hold for complex skill movements as well. Additionally, we hypothesized that the *mixed* training would accumulate the benefits of both types of learning, which could be reflected in rapid acquisition and good retention.

## Methods

### Participants

Sixty (n = 60) adults participated in the present study after giving their informed consent. All had normal or corrected-to-normal vision and were free from neurological/physical disorders or musculoskeletal injuries. The task in our study was the free throw skill in the basketball game. Participants were students who participated voluntarily, having all acquired the fundamental technique of the free throw during moderate practice in the past (college or secondary school). To homogenize our groups, former and current basketball players from French Federation basketball clubs or the university basketball team, as well as completely novice participants (who have never practiced basketball in the past), were excluded from the present study. This selection prevented a performance ceiling effect (i.e., no improvement due to a high-level initial accuracy) or demotivation due to a lack of success.

The participants were randomly assigned into four groups: the error-based group (n = 15, six females and nine males, mean age: 22.4 ± 1.3 years old), the reinforcement group (n = 15, seven females and eight males, mean age: 22.3 ± 2.1 years old), the mixed group (n = 15, eight females and seven males, mean age: 23.7 ± 1.7 years old), and the control group (n = 15, six females and nine males, mean age: 23.5 ± 1.4 years old). The regional ethic committee (Comité de Protection des Personnes—Région EST) approved the experimental design, which conformed to the standards set by the Declaration of Helsinki.

### Experimental device and procedure

**Figure 1:**
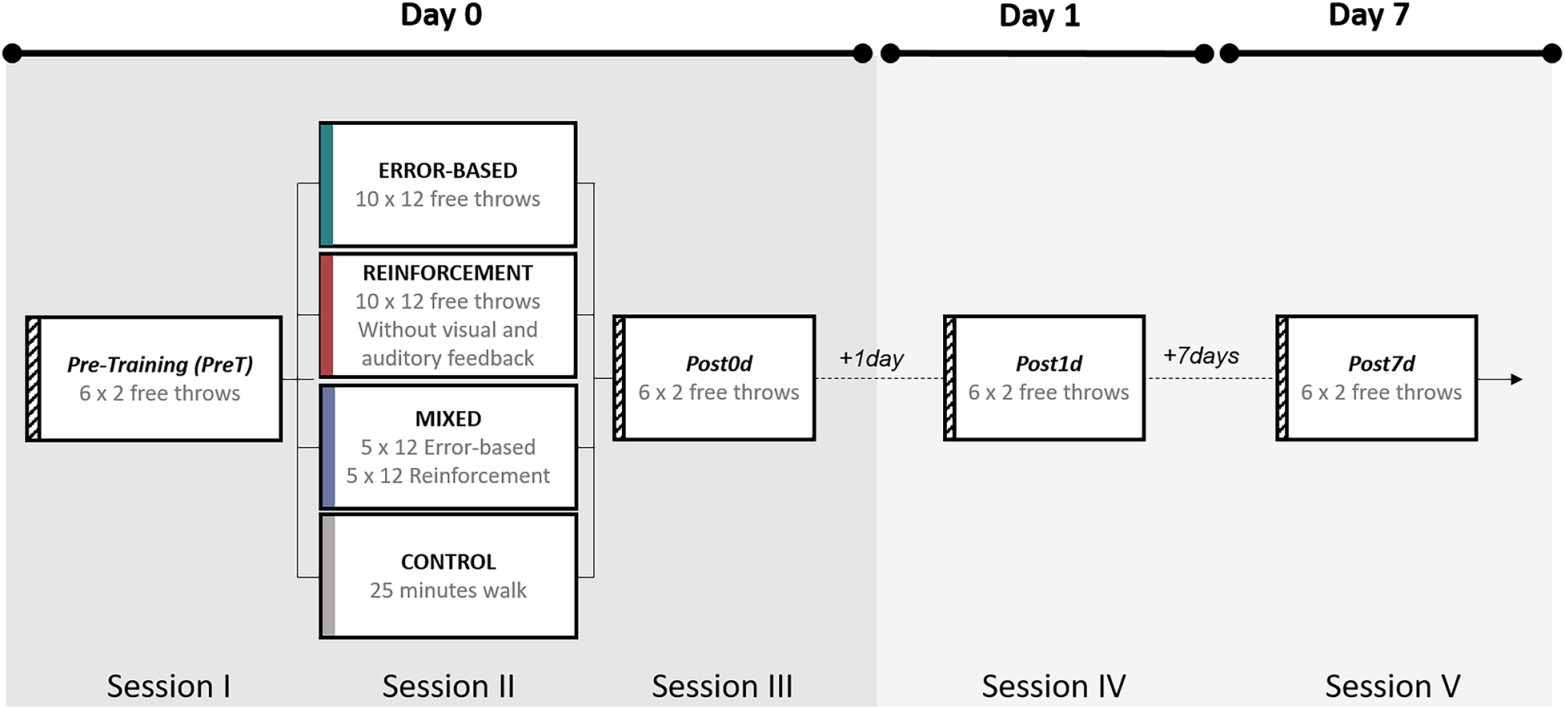
Experimental procedure. All participants carried out 6 blocks of 2 free throws in the Pre-Training test (PreT). For the training session, they were divided into four groups, and all, except those of the control group, carried out 120 free throws (i.e., 10 blocks of 12 free throws with 30 s of rest between blocks). During training, the error-based group received full sensory feedback, the reinforcement group received only binary feedback (success or failure), the mixed group received full sensory feedback followed by binary feedback, and the control group walked for 25 min, which corresponded to the training period of the other groups. Immediately (Post0d), one day (post1d), and seven days (post7d) after training, all participants carry out 12 free throws as in the PreT.

A classic basketball hoop was used. During the whole experiment, men used a size 7 ball, while women used a size 6 ball. The experiment included four measurements/tests (PreT, Post0d, Post1d, and Post7d) and one training session (Fig. 1). Note that during the experiments only the experimenter and each participant were present. In the Pre-Training test (PreT), all participants carried out 12 free throws, divided into 6 blocks of 2 free throws (to respect, as much as possible, the ecological conditions of the basketball game) with 15 s of rest between blocks. All had full sensory feedback regarding their free throw, but not any verbal feedback/instruction from the experimenter. The training session started 5 min after the PreT. All the participants, except those of the control group, carried out 120 free throws, 10 blocks of 12 free throws with 30 s of rest between blocks. The participants in the error-based learning group followed conventional free throw training. Specifically, they tried to score at each trial, always having sensory feedback on their performance to adapt/correct their free throw trial-by-trial^9,14,33^. The participants of the reinforcement group followed less conventional training, as they tried to score at each free throw without visual and auditory feedback on their performance. Precisely, the participants had a full vision of the basketball hoop at the beginning of each trial to adequately prepare their free throws. When they said ‘ready’, the experimenter occluded both vision (using a spectacle-mounted liquid crystal device: the PLATO Visual Occlusion Spectacles by Translucent Technologies) and audition (using noise-canceling headphones 700 by Bose) to private sensory feedback. After occlusion, all the participants performed the free shot in a short time interval (1–3 s). The experimenter verbally informed the participants whether the free throw was successful or not (i.e. binary feedback). No further information was provided regarding the trajectory of the ball. This absence of information obliged them to explore several solutions to find the optimal one^19,21,33^. The participants of the mixed group carried out the first 60 free throws with the same method as the error-based group and the next 60 free throws with the same method as the reinforcement group. Reinforcement learning was started after error-based learning to keep an ecological dynamic of motor learning^26^. In the control group, an active rest without manipulation of the ball replaced the training session. Specifically, the participants walked at a comfortable speed for 25 min, corresponding to the mean duration of the training session.

After the training session, all participants had 5 min of rest before the first post-training test (Post0d). The participants also accomplished two more post-training tests, one day (Post1d) and seven (Post7d) days later. The three post-training tests were performed in the same conditions as the PreT (6 blocks of 2 free throws, with 15 s of rest between blocks). In all tests, participants started with a 10-min warm-up, including specific basketball displacements and ten random shots excluding free throws.

### Data recording and analysis

For each session, the number of successful free throws was recorded as follows:

$$\frac{{Number\;of\;successful\;free\;throws{ }}}{12} \times 100$$

where 12 is the number of free throws in each session of pre-test (PreT) and post-test (Post0d, Post1d, and Post7d), as well as during each block of the training session.

Statistical analyses were performed using STATISTICA (8.0 version: Stat-Soft, Tulsa, OK) and JASP (0.16 version). The normal distribution of the data was verified by using the Shapiro–Wilk test. An a priori G*POWER analysis for total sample size estimation (parameters: Effect size f = 0.25; α = 0.05; power = 0.95; groups = 4; number of measurements = 4), indicated 13 participants per group (n = 52). The significance level was fixed at 0.05 (type I error) and power (type II error) was superior to 0.9 for all the statistical analyses. For the free throw accuracy, repeated measures (rm) ANOVA with *group* as a between-subjects factor (error-based, reinforcement, mixed, and control) and *session* as a within-subject factor (*PreT, Post0d, Post1d, and Post7d*) were applied. The effect size of (rm) ANOVA is reported as partial eta squared (η^2^) with small (0.01 ≤ η^2^ < 0.06), moderate (0.06 ≤ η^2^ < 0.14), and large effects (η^2^ ≥ 0.14). Fischer’s test was used for the post-hoc analysis, and the Bonferroni correction was applied to each *p*-value. The effect sizes of post hoc comparisons were reported as Cohen’s *d* with small (0.20 ≤ *d* < 0.50), moderate (0.50 ≤ *d* < 0.80), and large effects (*d* ≥ 0.80). In the absence of a significant difference, Bayesian equivalence analysis was performed with a region of practical equivalence ROPE = [− 0.1, 0.1] and a prior Cauchy scale of 0.707^34^.

## Results

### Main experiment

**Figure 2:**
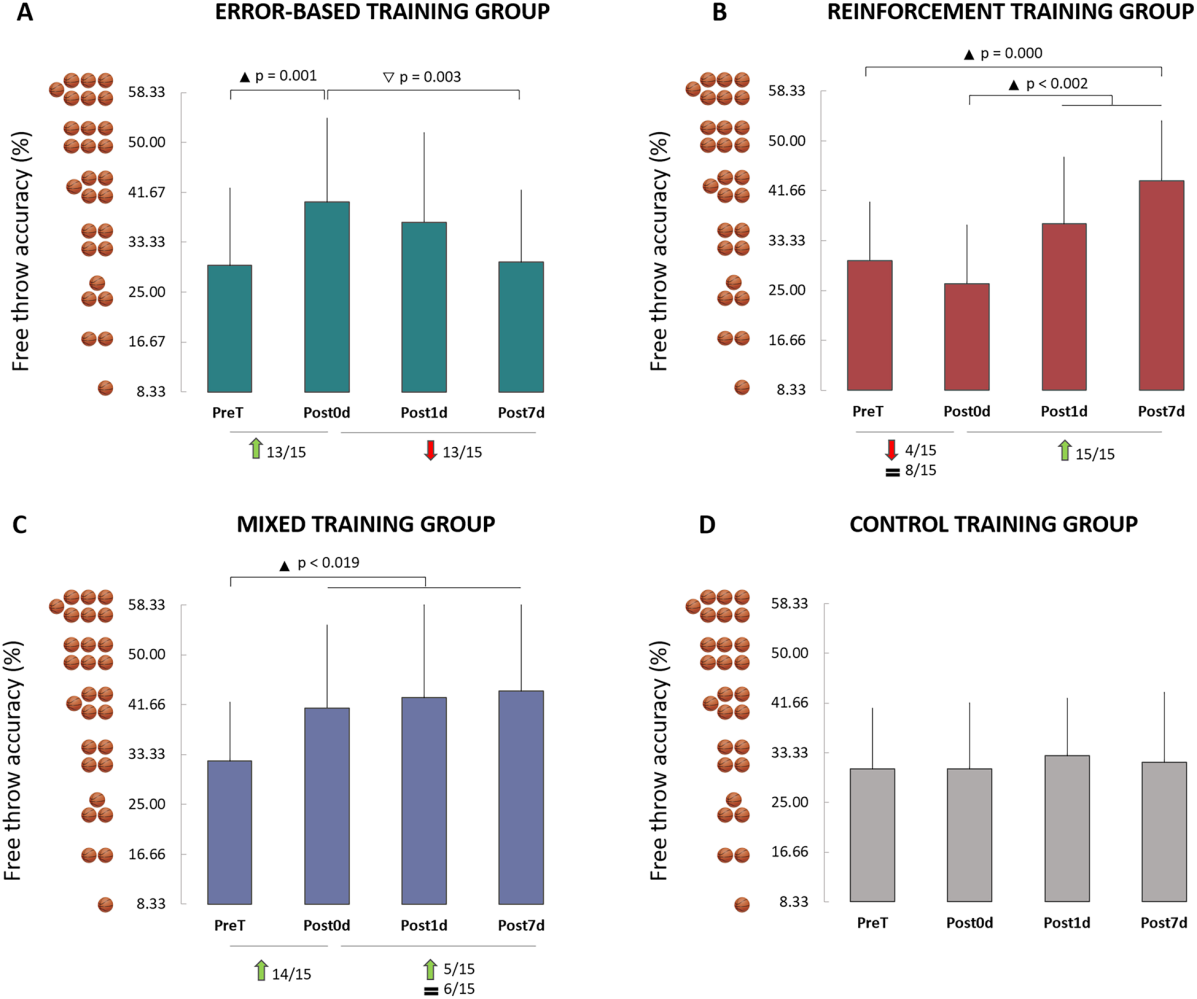
Average values (+ SD) of the free throw accuracy (%) for the four groups and the four tests. (**A**) Error-Based group. (**B**) Reinforcement group. (**C**) Mixed group. (**D**) Control group. Black triangles indicate significant improvement between tests, while the white triangle indicates significant deterioration. Green arrows, Red arrows, and the symbol ‘equal’ (=) indicate the number of participants who improved, decreased, or stabilized their freer throw accuracy, respectively. The basketballs near the vertical axis indicate the number of successful free throws.

Figure 2 illustrates the average values (+ SD, Standard Deviation) of the free throw accuracy (%) for the four groups and the four tests. ANOVA revealed a main significant effect of *session* (F_3,168_ = 9.97, *p* < 0.001, η^2^ = 0.15) and an interaction effect (*group* x *session*; F_9,168_ = 6.27, *p* < 0.001, η^2^ = 0.25) but not a main significant effect of *group* (F_3,56_ = 1.78, *p* < 0.162, η^2^ = 0.09).

The *post-hoc* analysis showed that initial accuracy (PreT) was not significantly different between groups (grand average 30.6%; i.e., 4 successful free throws out 12, for all comparisons: *p* = 1.000). Interestingly, free throw accuracy was improved after training accordingly to the specific learning method.

For the error-based group (Fig. 2A), an immediate positive effect of training was observed on free throw accuracy (PreT vs Post0d post hoc, *p* = 0.001, *d* = 1.80, 13/15 participants improved their accuracy). Note that accuracy enhancement after training lasted only for one day (Post0d vs Post1d post hoc*, p* = 1.000, *d* = 0.30). Seven days later, accuracy was deteriorated (Post0d vs Post7d post hoc, *p* = 0.003, *d* = 0.99; PreT vs Post7d post hoc, *p* = 1.000, *d* = 0.07). Thirteen out of fifteen (13/15) participants decreased their accuracy between Post0d and Post7d. The Bayesian equivalence test between PreT and Post7d showed the overlapping hypothesis Bayes Factor (BF^OH^_01_) was 3.70 and the non-overlapping hypothesis Bayes Factor (BF^NOH^_01_) was 5.02, meaning that the data are 3.7 times more likely to validate the null hypothesis than the alternative one and 5 times more likely to lie in the equivalence than in the non-equivalence region. These results showed moderate evidence that PreT and Post7d of the error-based group are equivalent, suggesting that the accuracy seems to return to the initial level 7 days after the training. This finding indicates that error-based training does not improve/stabilize new motor memories on a long-term scale.

An opposite result was found for the reinforcement group (Fig. 2B). Specifically, free throw accuracy was slightly decreased, although not significantly, after training (PreT vs Post0d post hoc, *p* = 0.996, *d* = 0.38; 4/15 participants decreased while 8/15 participants preserved their accuracy). Interestingly, accuracy was significantly improved one day after training (Post0d vs Post1d post hoc, *p* = 0.002, *d* = 0.84), as well as seven days after training (Post0d vs Post7d post hoc, *p* < 0.001, *d* = 1.21). Fifteen out of fifteen (15/15) participants increased their accuracy between Post0d and Post7d. This finding indicates that reinforcement training improves motor memory on a long-term scale.

The mixed group accumulated the advantages of the two previous training methods (Fig. 2C). There was a rapid improvement in free throw accuracy (PreT vs Post0d post hoc, *p* = 0.011, *d* = 0.70, 14/15 participants improved their accuracy) and long-term retention (Post0d vs Post1d post hoc, *p* = 1.000, *d* = 0.10; Post0d vs Post7d post hoc, *p* = 1.000, *d* = 0.17; Post1d vs Post7d post hoc, *p* = 1.000, *d* = 0.06). Eleven out of fifteen (11/15) participants stabilized or increased their accuracy between Post0d and Post7d.

Post hoc comparisons among groups at Post0d showed no significant differences in the free throw accuracy for the mixed and the error-based group (*p* = 1.000, *d* = 0.08) with weak evidence in favor of equivalence (Bayesian equivalence test: BF^OH^_01_ = 2.82; BF^NOH^_01_ = 3.42). However, a significant improvement was found in their accuracy compared to that of the reinforcement group (error-based vs reinforcement: *p* = 0.090, *d* = 1.14; mixed vs reinforcement: *p* = 0.004, *d* = 1.24). At Post7d, there were no significant differences in free throw accuracy for the mixed and the reinforcement group (post hoc: *p* = 1.000, *d* = 0.04) with weak evidence in favor of equivalence (Bayesian equivalence test: BF^OH^_01_ = 2.86; BF^NOH^_01_ = 3.49). However, a significant enhancement was found in their accuracy compared to that of the error-based group (reinforcement vs error-based, post hoc: *p* = 0.013, *d* = 1.20; mixed vs error-based for both, post hoc: *p* = 0.009, *d* = 0.94).

Finally, for the control group (Fig. 2D), the statistical analysis did not show any significant effect (in all, post hoc: *p* = 1.000, *d* < 0.28). The simple repetition of free throws during tests did not lead to any improvement in accuracy.

**Figure 3:**
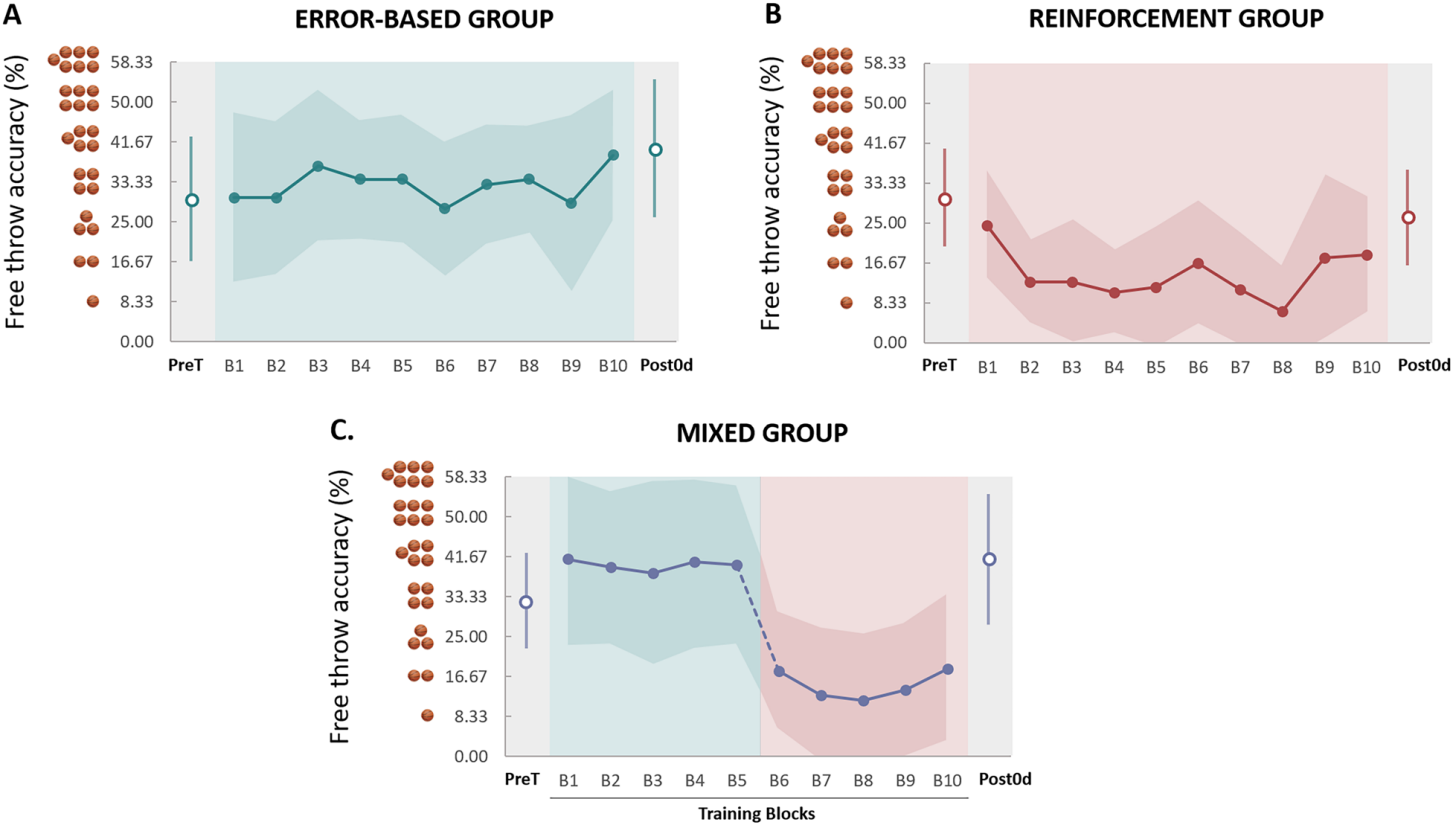
Block-by-block illustration of the free throw accuracy (%) during the training session (closed circles). Each block shows an average (+ SD) of 12 free throws. Open circles depict the free throw accuracy in pre-training (PreT) and post-training (Post0d) sessions. (**A**) Error-Based group. (**B**) Reinforcement group. (**C**) Mixed group. The broken line indicates the transition from error-based training to reinforcement training.

Figure 3 depicts block-by-block the evolution of free throw accuracy for the different training groups. It can be noted a regular improvement of free throw accuracy through training for the error-based group (Fig. 3A). For the reinforcement group, free throw accuracy initially decreased and then increased, remaining, however, below the pre-training values (Fig. 3B). This pattern can be explained by the specificity of training (i.e., without visual and auditory information of the movement), which forced the participants to explore different solutions, increasing thus the variability of their free shot.

### Control experiment

The better retention of free throw accuracy observed for the reinforcement group (Post7d) may be theoretically explained by the selection and reinforcement of the actions with a high success value and their consolidation into the long-term memory^18–20^. However, before attributing motor memory consolidation to this mechanism, one must exclude the possible effects of variability *per se*^35–37^. Indeed, error-based learning and reinforcement learning differ both in the nature of feedback and in the level of movement variability, which increases due to the exploration of different solutions^38^. The question, therefore, is whether an intentionally induced variability in an error-based learning context could improve and consolidate free throw accuracy as in reinforcement learning.

Our prediction is straightforward: if memory consolidation increases after a variable training in an error-based training context then motor memory consolidation must be attributed, at least partly, to variable training. On the contrary, reinforcement could be considered the essential mechanism for motor memory consolidation.

Fifteen new participants (seven females and eight males, mean age: 23.9 ± 2.2 years old) were included to perform the same task. All tests (PreT, Post0d, Post1d, and Post7d) were exactly similar to the main experiment, only the training session differed, during which the participants voluntarily reproduced the variability of free shots observed in the reinforcement group. Precisely, the participants of this group voluntarily fixed seven different goals: (1) successful free throws; (2) basketball 20–30 cm to the right without touching the hoop; (3) basketball 20–30 cm to the left without touching the hoop; (4) basketball 20–30 cm in front of the hoop without touching the hoop, (5) basketball touching the right side of the hoop; (6) basketball touching the left side of the hoop or (7) basketball touching the front of the hoop. These different goals were randomly distributed throughout the whole training; however, in each block of 12 free throws, participants executed, at least, once each goal.

**Figure 4:**
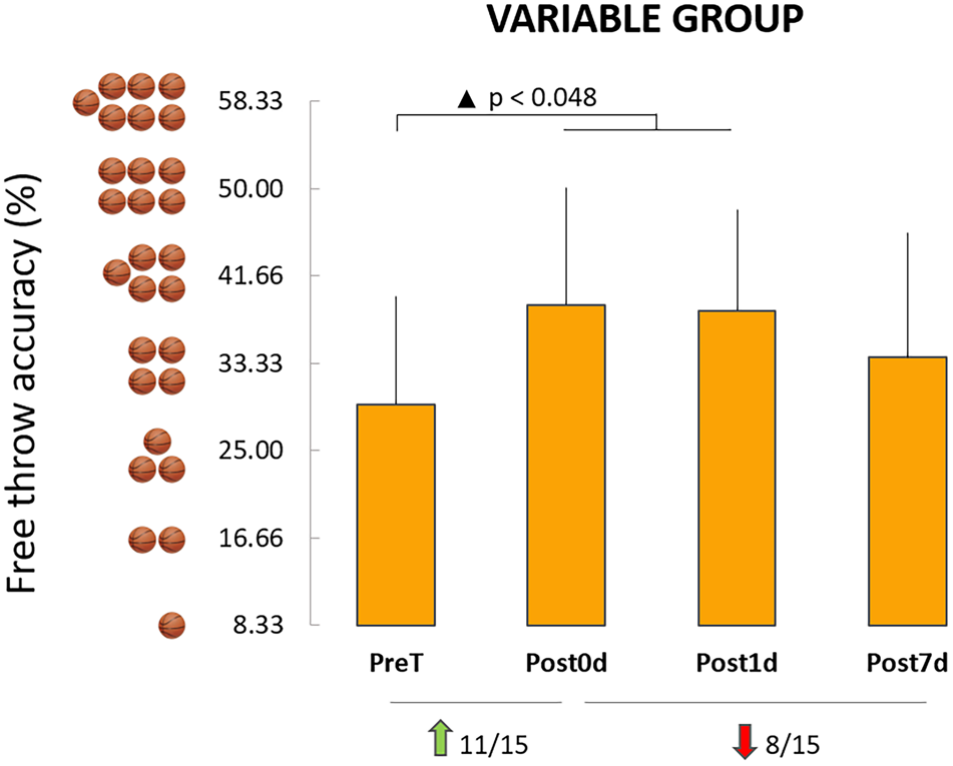
Average values (+ SD) of the free throw accuracy (%) for the variable group and the four tests. Black triangles indicate significant improvement between sessions. Green arrows, Red arrows indicate the number of participants who improved or decreased their free throw accuracy, respectively. The basketballs near the vertical axis indicate the number of successful free throws.

Figure 4 depicts the average values (+ SD) of the free throw accuracy (%) for the variable group. ANOVA revealed a significant effect of session (PreT, Post0d, Post1d, and Post7d, F_3,42_ = 3.74, *p* = 0.018, η^2^ = 0.21). Specifically, post hoc analysis showed that immediately after training there was a significant improvement in free throw accuracy (PreT vs Post0d, *p* = 0.033, *d* = 1.00; 11/15 participants increased their accuracy between PreT and Post0d) with a stabilization one day later (Post0d vs Post1d*, p* = 1.000; *d* = 0.05). Interestingly, seven days later, the variable group deteriorated its accuracy, although not significantly (Post1d vs Post7d, *p* = 1.000; 8/15 participants decreased their accuracy between Post1d and Post7d). The Bayesian equivalence test on Post1d and Post7d showed anecdotal evidence in favor of equivalence (BF^OH^_01_ = 2.46; BF^NOH^_01_ = 2.87). In addition, an independent samples t-test showed that the accuracy of the variable group at Post7d was significantly inferior to the reinforcement group (post hoc*: p* = 0.03, *d* = 0.81).

## Discussion

In the current study, the acquisition and retention of the basketball free throw shooting movement were examined following error-based, reinforcement, or mixed training. Better acquisition after error-based learning and better retention after reinforcement learning were found. A mixed training method founded first on error-based and then on reinforcement learning combined the advantages of both types of learning, namely strong acquisition and retention.

### Better acquisition with error-based learning than reinforcement learning

In line with previous studies^13,19,21^, an increase in accuracy immediately after error-based learning (Post0d) was observed. This finding can be explained by the theory of internal forward models^11^. Briefly, sensory prediction compared to actual sensory feedback leads to a remapping of the internal representation of the body (i.e., the internal forward model), improving thus the motor command^11,39^. It must be noted that target error, in addition to the sensory prediction error, also contributes to motor learning. This error informs the motor system of the difference between the goal of the task (i.e., the intention to have an accurate free throw) and the actual result (i.e., whether the free throw was successful or not)^40^. Therefore, both an implicit (i.e., sensorimotor prediction error) and an explicit (target error) process^40^ participated to free throw accuracy enhancement. In the current study, the contribution of each of these two errors to the free throw accuracy was not dissociated.

For the reinforcement group, no such improvement was found at Post0d. Instead, a rapid decrease was first observed in free throw accuracy followed by a slow and variable increase until returning to the baseline level (Fig. 3B). Greater variability is usually reported in studies involving reinforcement learning^19^. Indeed, motor variability denotes the exploration of various motor plans intending to find the successful movement^38^, which could explain why the reinforcement group did not immediately improve free throw accuracy. Note that in contrast with our findings, previous studies showed a significant increase in performance immediately after reinforcement training on simple motor tasks^19,41^. One possible explanation for this apparent contradiction could be the complexity of the motor task in our study^42,43^. Indeed, the basketball free throw shooting motion requires the coordination of several body parts as well as the control of the ball’s trajectory^44–46^. The control of many variables may cover the immediate effects of reinforcement learning^21,47^.

### Better retention with reinforcement learning than error-based learning

Despite the above-described difference in acquisition (PreT versus Post0d), the error-based and reinforcement groups showed similar accuracy one day after training (Post0d versus Post1d). Precisely, off-line enhancement (i.e., additional improvement in accuracy without additional practice^48^) was found after reinforcement learning versus a stabilization after error-based learning. Interestingly, the reinforcement group showed further off-line improvement in accuracy seven days after training (Post7d), whereas accuracy for the error-based group deteriorated, returning to initial performance. The passage of time after reinforcement learning improved accuracy by facilitating the retention and selection of the successful movement. These results are consistent with previous behavioral findings on simple motor skills^20,21,32,49^ and suggest that reinforcement learning leads to a stronger memory trace than error-based learning. Several studies have reported that better consolidation after reinforcement training was due to the induction of dopamine-dependent long-term potentiation^41,50^.

Our control experiment showed that an intended-induced variability in an error-based learning context (i.e., variable training) was not sufficient to consolidate motor memory. This finding supports the specific and essential mechanism of reinforcement to induce a stronger memory trace. Nevertheless, new investigations are necessary to understand the consolidation of the memory trace despite no immediate improvements in accuracy after reinforcement training. Walker et al. showed no correlation between the gain quantity immediately after motor learning and the off-line learning obtained after a period of rest, suggesting that memory consolidation after training may arise from a different mechanism than its acquisition^51^.

The motor memory forgetting observed for the error-based group strongly corroborates previous findings^32,41^. It is possible that the adjustment of the motor controller, in an error-based learning context, is more fragile compared to the formation of a new motor controller in a reinforcement learning context^52^. Indeed, immediately after the acquisition process, the motor memory may be deteriorated by proactive or retroactive interferences (as other memories)^53–55^. In the case of error-based learning, the original skill may compete with the new adjustment, as a proactive interference, leading to forgetting and returning to the initial level^32^. However, further investigation is needed to provide a better understanding of the mechanisms contributing to this forgetting.

It must be noted that both sensory and reward feedbacks are available during training for the error-based group. Indeed, the sensory feedback that leads to identifying the error vector (error signal) also acts as a reward signal (the participant sees whether the free throw is successful or not). This double information, however, is not sufficient for the retention of free throw accuracy on a long-term scale. It appears, therefore, that if sensory feedback and reward feedback are both available during training, error-based learning overrides reward-based learning, as is the case between an implicit motor plan and an explicit motor strategy during sensorimotor adaptation^56,57^.

### The combination of error-based and reinforcement learning

For the mixed group (i.e., sensory feedback first and then reward feedback only), an increase in free throw accuracy immediately after training was observed, which stabilized one (Post1d) and seven days (Post7d) later. This result indicates that the mixed group combined the benefits of both error-based and reinforcement learning, with strong acquisition and retention, respectively. Consistent with our study, previous investigations, using a visuomotor adaptation task, showed that motor learning can be stabilized when it was followed by periods of reward training^32^. Such a sequential learning scheme seems optimal, in which error-based learning does not override reward-based learning (see the previous section). Indeed, Huang et al. showed that in a simple motor learning task, reward feedback would be considered once the error was significantly reduced, with error-based learning occurring earlier in training than reinforcement learning^26^. Our mixed group showed that the removed sensory feedback in the second part of training promotes the retention of complex motor movement.

### Implications and perspectives

The current study tried to provide a more complete comprehensive understanding of the effects of error-based and reinforcement learning on the acquisition and retention of a whole-body movement demanding high accuracy. A mixed training method, combining both error-based and reinforcement learning, clearly gives the best learning outcome. Our results may help to design effective interventions in physical education programs and sports like basketball academies. Indeed, our findings show that a successful training method to rapidly improve a motor skill is the error-based method. This method, using sensory feedback, helps the learner to develop a better motor repertoire through the improvement of the motor prediction process. This is a powerful method with rapid improvement; it has, however, the disadvantage of rapid forgetting. If a coach/teacher wants to consolidate a motor skill, then reinforcement learning, using reward feedback, must be added to the learning process. This method alone may be tough at the beginning of a pedagogical or sports program because motor performance improvement is slow. For that reason, we suggest that a mixed method, combining sensory and reward feedback, is the best solution to learn a new motor skill, especially during early physical education or sports programs. New investigations are necessary to better understand how to combine the mixed method, i.e., within a learning session, separately, or both, to better improve motor skills. In addition, it would be interesting to extend this study to high-skilled basketball players to find the optimal training method to further improve skill.

## Data Availability

The datasets generated during and analyzed during the current study are available from the corresponding author upon reasonable request.
